# The family Oestridae in Egypt and Saudi Arabia (Diptera, Oestroidea)

**DOI:** 10.3897/zookeys.947.52317

**Published:** 2020-06-08

**Authors:** Magdi S. A. El-Hawagry, Mahmoud S. Abdel-Dayem, Hathal M. Al Dhafer

**Affiliations:** 1 Department of Entomology, Faculty of Science, Cairo University, Egypt Cairo University Giza Egypt; 2 College of Food and Agricultural Sciences, King Saud University, Riyadh, the Kingdom of Saudi Arabia King Saud University Riyadh Saudi Arabia

**Keywords:** Activity periods, bot flies, distribution, gad flies, heel flies, hosts, localities, parasites, warble flies

## Abstract

All known taxa of the family Oestridae (superfamily Oestroidea) in both Egypt and Saudi Arabia are systematically catalogued herein. Three oestrid subfamilies have been recorded in Saudi Arabia and/or Egypt by six genera: *Gasterophilus* (Gasterophilinae), *Hypoderma*, *Przhevalskiana* (Hypodermatinae), *Cephalopina*, *Oestrus*, and *Rhinoestrus* (Oestrinae). Five *Gasterophilus* spp. have been recorded in Egypt, namely, *G. haemorrhoidalis* (Linnaeus), *G. intestinalis* (De Geer), *G. nasalis* (Linnaeus), *G. nigricornis* (Loew), and *G. pecorum* (Fabricius). Only two of these species have also been recorded in Saudi Arabia, namely: *G. intestinalis* (De Geer) and *G. nasalis* (Linnaeus). The subfamily Hypodermatinae is represented in the two countries by only four species in two genera, namely, *H. bovis* (Linnaeus) and *H. desertorum* Brauer (in Egypt only), and *H. lineatum* (Villers) (in Saudi Arabia only) and *Przhevalskiana silenus* (Brauer) (in both countries). The subfamily Oestrinae is represented by two widely distributed species in both countries, namely, *C. titillator* (Clark) and *O. ovis* (L.), in addition to another species represented in Egypt only, *R. purpureus* (Brauer). For each species, synonymies, type localities, distribution, Egyptian and Saudi Arabian localities with coordinates, and collection dates are presented.

## Introduction

The Oestridae are a family within the superfamily Oestroidea, together with the families Calliphoridae, Rhiniidae, Sarcophagidae, Mystacinobiidae, Tachinidae, and Rhinophoridae (Pape et al. 2011). These families, except for Calliphoridae, are monophyletic, and the concept of Oestridae as a monophyletic family within the Oestroidea has been clearly established (Pape 1992; Pape 2001; Pape and Arnaud Jr 2001; Marinho et al. 2012).

Flies of the family Oestridae are large robust flies, with hair-like setae or soft setulae, without stout setae, mostly bee- or wasp-like, without vibrissae, and with reduced mouthparts (Marshall et al. 2017). They are commonly known as bot flies, warble flies, heel flies, and gad flies (Mote 1928; Saini and Sankhala 2015). Several species of these flies have significant medical and veterinary importance because of their mammal-parasitizing habits; thus, they receive substantial attention from applied entomologists, wildlife ecologists, and assuredly from taxonomists (Pape 2001).

Bot flies were formerly classified into four families: Cuterebridae, Gasterophilidae, Hypodermatidae, and Oestridae. However, they are conveniently treated now as a single family, Oestridae, including the former families as subfamilies, namely: Cuterebrinae, Gasterophilinae, Hypodermatinae, and Oestrinae (Wood 1987; Pape 1992; Pape 2001). All these subfamilies, except the first, are represented in Saudi Arabia and/or Egypt by six genera (Table 1): *Gasterophilus* (Gasterophilinae), *Hypoderma*, *Przhevalskiana* (Hypodermatinae), *Cephalopina*, *Oestrus* and *Rhinoestrus* (Oestrinae) (Steyskal and El-Bialy 1967; Büttiker and Zumpt 1982).

**Table 1.** Oestrid species recorded from Egypt and Saudi Arabia (* = recorded, x = not recorded).

Species	Egypt	Saudi Arabia
**Subfamily Gasterophilinae**		
*Gasterophilus haemorrhoidalis* (Linnaeus, 1758)	*	x
*Gasterophilus intestinalis* (De Geer, 1776)	*	*
*Gasterophilus nasalis* (Linnaeus, 1758)	*	*
*Gasterophilus nigricornis* (Loew, 1863)	*	x
*Gasterophilus pecorum* (Fabricius, 1794)	*	x
**Subfamily Hypodermatinae**		
*Hypoderma bovis* (Linnaeus, 1758)	*	x
*Hypoderma desertorum* Brauer, 1897	*	x
*Hypoderma lineatum* (Villers, 1789)	x	*
*Przhevalskiana silenus* (Brauer, 1858)	*	*
**Subfamily Oestrinae**		
*Cephalopina titillator* (Clark, 1816)	*	*
*Oestrus ovis* (Linnaeus, 1758)	*	*
*Rhinoestrus purpureus* (Brauer, 1858)	*	x

Larvae of the genus *Gasterophilus* are common obligatory endoparasites of the alimentary tract of equines (*Equus* spp.) including horses, donkeys, and zebras in the family Equidae (Abdel Rahman et al. 2018). They can also affect other animals, such as rhinoceroses, lions, cows, sheep, goats, and even were recorded in a human infant (Royce et al. 1999). These larvae cause gastrointestinal myiasis leading to gastrointestinal ulcerations, gut obstructions or volvulus, rectal prolapses, anemia, diarrhea, and other digestive disorders (Hoseini et al. 2017). Species of the genus *Gasterophilus* have become near cosmopolitan because their distribution coincides with that of their domesticated hosts (Li et al. 2019a). Six *Gasterophilus* spp. have been recorded from the Old World (Zumpt 1965; Soós and Minar 1986a). Five of these have been recorded in Egypt, namely, *G. haemorrhoidalis* (Linnaeus), *G. intestinalis* (De Geer), *G. nasalis* (Linnaeus), *G. nigricornis* (Loew), and *G. pecorum* (Fabricius) (Steyskal and El-Bialy 1967, Soós and Minar 1986a). Only two have also been recorded from Saudi Arabia, namely: *G. intestinalis* and *G. nasalis* (Abu-Thuraya 1982; Büttiker and Zumpt 1982; Abu-Zoherah et al. 1993; Al-Ahmadi and Salem 1999).

The subfamily Hypodermatinae is represented in both Egypt and Saudi Arabia by only four species in two genera, namely, *H. bovis* (Linnaeus) and *H. desertorum* Brauer (in Egypt only), and *H. lineatum* (Villers) and *P. silenus* (Brauer) (in both Egypt and Saudi Arabia) (Steyskal and El-Bialy 1967; Büttiker and Zumpt 1982; Soós and Minar 1986b; El-Azzazy 1997; Morsy et al. 1998). The common and best known subcutaneous myiasis in domesticated and wild ruminants called bovine hypodermosis is caused by larvae of *Hypoderma* species across the Old World (Boulard 2002). This disease is endemic in livestock, including cattle, buffaloes, goats, sheep, and deer. Hypodermosis results in a severe decline in the production of meat and milk and depreciation in hide quality from holes and other flaws caused by *Hypoderma* larvae (Hall and Wall 1995). The larvae of *P. silenus* (goat warble fly) are known to cause subcutaneous myiasis distinguished by nodules on the back of goats and sheep. This myiasis causes severe economic problems to the livestock industry, including abortion and reduction in the body weight, fertility, and dairy production of the infested animals, in addition to a reduction in the quality of the hides and wool of the animal (Liakos 1986; El-Azzazy 1997).

Flies in the subfamily Oestrinae are known as nasopharyngeal bot flies; they are host specific and cause obligatory myiasis in many animal species. Their obligatory parasitic larvae are known to cause nasopharyngeal myiases giving rise to respiratory problems, rhinitis, irritation, purulent mucous exudates, and nasal discharge (Catts and Mullen 2002; Otranto et al. 2003). Two oestrine species are widely distributed in both Egypt and Saudi Arabia, namely, *O. ovis* (sheep nasal bot fly) and *C. titillator* (camel nasal bot fly), which cause economic damage in the animal husbandry industry (Abu-Thuraya 1982; Büttiker and Zumpt 1982; Zayed 1998; Alahmed 2002). Another oestrine species, *R. purpureus* (equine nasal bot fly), is represented in Egypt and causes a parasitic disease in horses and donkeys called rhinoestrosis, which is characterized by clinical signs ranging from inflammation to coughing, sneezing, and dyspnea (Otranto 2004; Hilali et al. 2015).

**Figure 1.**
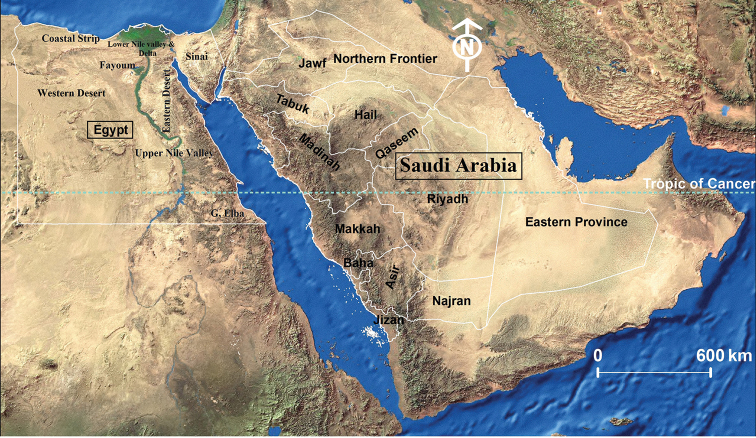
A satellite map of Egypt and Saudi Arabia.

Egypt and Saudi Arabia are two neighboring Middle Eastern countries separated by the Red Sea and the Gulf of Aqaba (Fig. 1). They are biogeographically comparable being located at the junction of the Palearctic and the Afrotropical Realms (Wallace 1876; Hölzel 1998; El-Hawagry and Gilbert 2014).

An arid desert climate prevails in both countries, with the exception of small strip of the Mediterranean coastline in Egypt and the Asir Highlands along the Red Sea coast of Saudi Arabia. The climate in both countries is characterized by hot summer and a mild winter. From north to south across Egypt, three general climatic zones may be distinguished (Ullrich 1996): The Mediterranean coast zone with 70–200 mm annual precipitation and mean temperature ranging from 9.4 °C in January to 29.7 °C in July; the middle zone with 29N as its latitudinal boundary, with less than 1 mm (Siwa Oasis) to 35 mm (Cairo) annual precipitation, and has only slightly higher temperature than the Mediterranean coast zone and the third zone is the upper Egypt, where rainfall is scant and capricious, ranging from 3 mm (Aswan) to none, with mean temperature (at Aswan) ranging from 9.3 °C in January to 41.8 °C in July. In general, the rainfall is low in the most Egyptian areas and deserts (<80 mm annually). Only the Mediterranean coastal strip from Salloum to Alexandria, Gebel Elba in the extreme southeast, and the mountains of southern Sinai receive higher and less erratic rainfall (ca 200 mm annually). In Saudi Arabia, the average annual temperature is 25.2 °C, the average high temperature is about 37.8 °C during summer (June to August) and is about 11.1 °C during winter (December to February). It is cool, with frost and snow may occur in the Asir Highlands during winter. The precipitation is also low throughout the country (<100 mm). It is more than 480 mm in the highlands of Asir; however, a decade may pass with no precipitation at all in the Rub’ al Khali (Empty Quarter) in the southeastern Saudi Arabia (Almazroui 2011).

Efflatoun Bey, often called the “father of Egyptian entomology”, comprehensively surveyed the Diptera of Egypt and established big collections of flies pinned and preserved in three Egyptian museums in Cairo University, Ministry of Agriculture, and Entomological Society of Egypt. The oestrid specimens in these collections are considered in the present study.

During the nineteenth century, two species of subfamily Oestrinae, *Oestrus maculatus* Wiedemann, 1830 and *O. libycus* Clark, 1843, originally described from Egypt have been later synonymized with *Cephalopina titillator*. Then Brauer (1897) has described *Hypoderma desertorum* from Helwan (Cairo), Egypt.

No systematic studies on bot flies have been previously conducted in Egypt. Only a list of species of dipterous families in Egypt was published by Steyskal and El-Bialy (1967), where 1,339 species have been listed, including 10 oestrid species (treated as Gasterophilidae and Oestridae). The list involved only family names with a list of species within each family, without any other taxonomic or faunistic data. Subsequently, between 1987 and 2018, the species prevalence and infestation by oestrids have been received attention by entomologists and veterinarians, but no study has been carried out to explore the national prevalence of this group. The infestation of donkeys by *Gastrophilus* and *Rhinoestrus* species has been investigated in the slaughterhouse of the National Cairo Circus and in Giza Zoo abattoir by Hilali et al. (2015) and Attia et al. (2018). In sheep, the infestation by maggots of *Oestrus ovis* in Cairo and *Przhevalskiana silenus* in Sinai has been studied by Amin et al. (1997) and Morsy et al. (1998), respectively. Two studies have been conducted to illustrate the morphological characterization of larval stage of *Gasterophilus* species infest stomach of donkeys (El-Bakry and Fadly 2014, Abdel Rahman et al. 2018).

Although documentation of biological diversity in Saudi Arabia began in the second half of the 1960s, the first traces of the Saudi Arabian oestrid flies are found in a work dated 1982, as five species, *Cephalopina titillator*, *Gasterophilus intestinalis*, *G. nasalis*, *Hypoderma lineatum*, and *Oestrus ovis* have been mentioned from Riyadh Region (Büttiker and Zumpt 1982). In the same year, a book on the agricultural pests in the Kingdom of Saudi Arabia has been published (Abu Thuraya 1982). This book has documented four species *C. titillator*, *G. intestinalis*, *G. nasalis*, and *O. ovis*. El-Azzazy (1997) reported the larvae of the goat warble fly, *Przhevalskiana silenus*, on the backs of goat carcasses at the Jeddah abattoir (Makkah Region) for the first time. Between 1988–2018, entomological, medical and veterinary works have been published, but most of these studies were carried out at provincial scale. The ocular myiasis in man caused by the sheep bot fly *O. ovis* has been firstly reported in Saudi Arabia from Abha (Asir Region) by Omar et al. (1988). The prevalence variation of *C. titillator* infesting dromedary camels has been studied in the Eastern Province (Fatani and Hilali 1994), Jeddah (Gadallah and Bosly 2006) and Riyadh (Alahmed 2002). Also, the prevalence of *O. ovis* infesting sheep has been investigated in Asir (Kenawy et al. 2014), Jazan (Bosly 2013), Jeddah (Alikhan et al. 2018) and Riyadh (Alahmed 2000). Akhter et al. (2000) report two cases of cutaneous infestation in a man and a woman caused by *Dermatobia hominis* in Taif, Saudi Arabia. This record is doubtful as *D. hominis* is native to the Americas, and the species was identified only from larvae.

This study is one in a series of studies planned to catalogue the superfamily Oestroidea in Egypt and Saudi Arabia. Two papers in this series have already been published (El-Hawagry 2018; El-Hawagry and El-Azab 2019).

## Materials and methods

The present data were gathered from some adult specimens collected and pinned by the authors from different Egyptian and Saudi Arabian localities, in addition to adult specimens pinned and preserved in Efflatoun Bey’s collection, Department of Entomology, Faculty of Science, Cairo University, Egypt (EFC); the Ministry of Agriculture Collection, Plant Protection Research Institute, Dokki, Giza, Egypt (PPDD), and the King Saud University Museum of Arthropods, Riyadh, Saudi Arabia (KSMA). A great deal of biological, faunistic, and taxonomic information, including synonymies, distribution, collection localities, and dates were also obtained from relevant literature.

This study catalogues all known taxa of the family Oestridae recorded from Egypt and Saudi Arabia. Subfamilies are arranged phylogenetically according to Pape (2001). Genera and species within subfamilies are arranged alphabetically. Synonyms comprised all available and unavailable names of genera and species are listed chronologically.

Family-group and genus-group names are written in bold uppercase letters and left-justified, with the genus-group names italicized. The genus-group names are listed again and left-justified under the headings, and written in bold italicized letters, with the first letter in uppercase and the remaining letters in lowercase, followed by the author, year, journal, and pages. Type species for each genus is given at the end, followed by the method by which it was fixed. Species names are left-justified as well, and written in bold italicized letters. Names of taxonomically valid species (senior synonyms) are listed again, combined with their original genera and left-justified under the headings followed by the author, year, journal, and pages. Synonyms of genera and species are listed in chronological order and written in regular italicized letters, followed by the author, year, journal, and pages as in senior taxa. The type locality for each species, including both senior and junior synonyms, is provided from the original descriptions. World distribution of each species based on relevant literature is listed alphabetically. The concept of Kirk-Spriggs and Sinclair (2017) regarding the boundaries between the Palearctic and Afrotropical realms is considered herein. Exceptions are the southwestern part of Saudi Arabia, south to the Tropic of Cancer and Gebel Elba, the southeastern triangle of Egypt, which are considered herein as Afrotropical (Sclater 1858; Wallace 1876; Ghazanfar and Fisher 1998; El-Hawagry and Gilbert 2014; Al Dhafer and El-Hawagry 2016; El-Hawagry 2017; El-Hawagry et al. 2018). The collection localities and dates in both Egypt and Saudi Arabia are given in tables to provide the local distribution and activity periods of oestrid flies. Localities within each Egyptian ecological zone and Saudi Arabian region are arranged in alphabetical order. The recording method, e.g., literature, museum material, and collected material are provided. Coordinates of each locality are mostly given, and distribution maps for species are provided using ArcMap 10.4.

Abbreviations used:

**AF** Afrotropical Realm

**AU** Australasian Realm

**EFC** Collection of the Department of Entomology, Faculty of Science, Cairo University, Egypt (Efflatoun’s collection)

**KSA** Kingdom of Saudi Arabia

**KSMA** King Saud University Museum of Arthropods, Riyadh, Saudi Arabia

**Is** Island

**MCCB** Museum of Community College, Al-Baha University, KSA

**MSHC** Personal collection M. El-Hawagry

**NE** Nearctic Realm

**NEO** Neotropical Realm

**OR** Oriental Realm

**PA** Palearctic Realm

**PPDD** Collection of the Plant Protection Research Institute, Ministry of Agriculture, Dokki, Giza, Egypt

**St.** Saint

**USA** United States of America

## Catalogue of the family Oestridae in Egypt and Saudi Arabia

**Order: Diptera**

**Suborder: Cyclorrhapha**

**Superfamily: Oestroidea**

**Family Oestridae**

**Subfamily Gasterophilinae**

### Genus Gasterophilus

Taxon classificationAnimaliaDipteraOestridae

Leach, 1817

55AF2635-D7D0-5BD5-B866-A5E858DCA236

Gasterophilus Leach, 1817: 2. Type species: Oestrus equi Clark, 1797 (= Oestrus intestinalis De Geer, 1776), by subsequent designation of Curtis, 1826: 146.
Gastrus Meigen, 1824: 174. Type species: Oestrus intestinalis De Geer, 1776, by subsequent designation of Coquillett, 1910: 546.
Gastrophilus Agassiz, 1846: 160. Invalid emendation of Gasterophilus.
Enteromyza Rondani, 1857: 20. Unnecessary replacement name for Gasterophilus.
Rhinogastrophilus Townsend, 1918: 152. Type species: Oestrus nasalis Linnaeus, 1758, by original designation.
Enteromyia Enderlein, 1934: 425. Type species: Oestrus haemorrhoidalis Linnaeus, 1758, by original designation.
Stomachobia Enderlein, 1934: 425. Type species: Oestrus pecorum Fabricius, 1794, by original designation.
Haemorrhoestrus Townsend, 1934: 406. Type species: Oestrus haemorrhoidalis Linnaeus, 1758, by original designation.
Progastrophilus Townsend, 1934: 406. Type species: Oestrus pecorum Fabricius, 1794, by original designation.

### Gasterophilus haemorrhoidalis

Taxon classificationAnimaliaDipteraOestridae

(Linnaeus, 1758)

746A2A68-A481-5D4B-AA9F-CA27F6D4A66F

Oestrus haemorrhoidalis Linnaeus, 1758: 584. Type localities: Probably Sweden, Germany, and France (see Li et al. 2019b).
Oestrus salutiferus Clark, 1816: 3. Type locality: England.
Oestrus duodenalis Schwab, 1840: 35. Type locality: Europe.
Gastrophilus pallens Bigot, 1884: 4. Type locality: Sudan (Suakin).
Gasterophilus pseudohaemorrhoidalis Gedoelst, 1923: 272. Type localities: Eritrea (Asmara); Republic of the Congo, Katanga Province (Biano), and Zambia.
Oestrus hemorrhoidalis Clark, 1815: 71. Incorrect subsequent spelling of haemorrhoidalis Linnaeus, 1758.
Oestrus hemorroidalis Guérin-Méneville, 1827: 96. Incorrect subsequent spelling of haemorrhoidalis Linnaeus, 1758.
Oestrus aemorrhoidalis Rondani, 1857: 21. Incorrect subsequent spelling of haemorrhoidalis Linnaeus, 1758.

#### Common name.

Nose bot fly or Lip bot fly.

#### Distribution.

AF: Burkina Faso, Democratic Republic of the Congo, Eritrea, Ethiopia, Kenya, Namibia, Republic of the Congo, Senegal, South Africa, Sudan, Tanzania, Zambia. AU: Australia, Hawaii, New Zealand, Tasmania. NE: Canada (Alberta, British Columbia, Manitoba, Saskatchewan), Mexico, USA (widespread). NEO: Argentina, Venezuela. OR: India. PA: Widespread. (see Soós and Minar 1986a; Kettle 1995; Li et al. 2019b).

#### Localities, hosts, and dates of collection.

See Table 2 and Figure 3.

**Table 2.** Localities, hosts, and dates of collection of *G. haemorrhoidalis*.

Country	Zone or Region	Locality	Coordinates	Host/s	Months of collection	Reference
Egypt	Coastal Strip	Alexandria	31.203358N, 29.917285E	mules and donkeys (from stomachs)	from October to April	El-Bakry and Fadly (2014)

### Gasterophilus intestinalis

Taxon classificationAnimaliaDipteraOestridae

(De Geer, 1776)

37ECC6A4-101F-5BD8-A7BE-676D5FD25891

Fig. 2a

Oestrus intestinalis De Geer, 1776: 292. Type locality: Sweden.
Oestrus equi Clark, 1797: 298. Preoccupied by Fabricius, 1787. Type locality: England.
Oestrus gastricus major Schwab, 1840: 31. Unavailable name.
Oestrus bengalensis Macquart, 1843: 182. Type localities: Bangladesh and India.
Oestrus gastrophilus Gistel, 1848: 153. Type locality: Probably Germany.
Oestrus schwabianus Gistel, 1848: 153. Type locality: Probably Germany (Bavaria).
Gastrophilus equi var. asininus Brauer, 1863: 71. Type localities: Egypt and Sudan (“Egypten” & “Nubien”).
Gastrophilus aequi : Brauer 1863: 28. Incorrect subsequent spelling of equi Clark, 1797.
Gasterophilus magnicornis Bezzi, 1916: 29. Type locality: Eritrea.

#### Common name.

Horse bot fly.

#### Distribution.

AF: Burkina Faso, Chad, Eritrea, Ethiopia, Ghana, Kenya, Morocco, Nigeria, Republic of the Congo, Senegal, South Africa, St. Helena, Sudan, Tanzania, United Arab Emirates. AU: Australia (New South Wales, Norfolk Is, Tasmania), Hawaii, New Zealand. NE: Canada (Alberta, British Columbia, Manitoba, New Brunswick, Ontario, Quebec, Saskatchewan), Mexico (Aguascalientes, Chiapas), USA (widespread). NEO: Argentina, Brazil (Rio Grande do Sul), Chile (Bío Bío Region), Jamaica, Venezuela. OR: India. PA: Widespread. (see Soós and Minar 1986a; Kettle 1995; Li et al. 2019b).

#### Localities, hosts, and dates of collection.

See Table 3 and Figure 3.

**Table 3.** Localities, hosts, and dates of collection of *G. intestinalis*.

Country	Zone or Region	Locality	Coordinates	Host/s	Months of collection	Reference
Egypt	Coastal Strip	Alexandria	31.203358N, 29.917285E	mules and donkeys (from stomachs)	from October to April	El-Bakry and Fadly (2014)
	Lower Nile Valley & Delta	Cairo (at slaughterhouse of the National Cairo Circus)	30.122446N, 31.360598E	donkeys	throughout the year	Hilali et al. (1987)
		Cairo (at Cairo Manure Co.)	30.102160N, 31.253994E	mules and donkeys (from stomachs)	April to December	museum material (see material examined)
		Cairo (abattoir)	30.040022N, 31.244248E	donkeys (from stomachs)	June	museum material (see material examined)
		Giza (Giza Zoo)	30.027973N, 31.215963E	donkeys (from stomachs)	throughout the year	Abdel Rahman et al. (2018); Attia et al. (2018)
KSA	widespread in all regions, especially abundant in Al-Ehsaa, El-Kharj and Riyadh	Al-Ehsaa	25.388528N, 49.596223E	donkeys and horses (from stomachs)	March to September	Abu-Thuraya (1982)
		El-Kharj	24.148402N, 47.305011E	donkeys and horses (from stomachs)	March to September	
		Riyadh (near slaughterhouse)	24.578977N, 46.736175E	from dead domestic horse	March	Büttiker and Zumpt (1982)

#### Material examined.

Egypt • 1 male; Cairo Manure Co.; 30.102160N, 31.253994E; 13.Nov.1924; from the stomach of a donkey; EFC • 1 male; same data as for preceding; 22.Apr.1930 • 1 male; same data as for preceding; 23.Nov.1930 • 1 female; same data as for preceding; 29.Oct.1924; PPDD • 1 ?male; same data as for preceding; Cairo abattoir; 30.040022N, 31.244248E; 7.Jun.1924.

**Figure 2.**
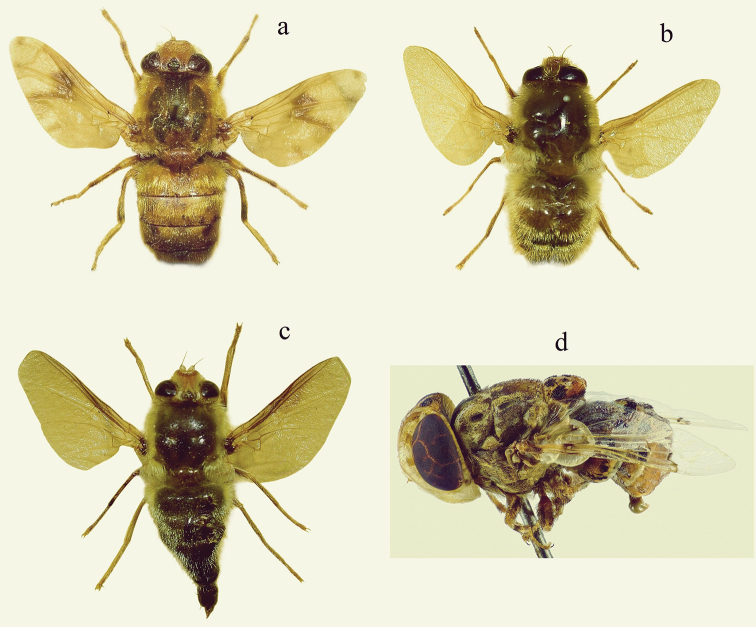
**a***Gasterophilus intestinalis* (habitus, dorsal) **b***G. nasalis* (habitus, dorsal) **c***G. nigricornis* (habitus, dorsal) **d***Cephalopina titillator* (habitus, lateral).

**Figure 3.**
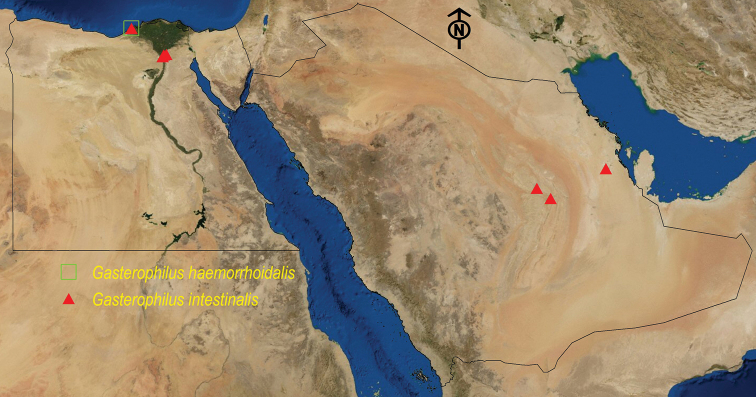
Distribution map of *G. haemorrhoidalis* and *G. intestinalis*.

### Gasterophilus nasalis

Taxon classificationAnimaliaDipteraOestridae

(Linnaeus, 1758)

368E6F1E-0C32-5E69-9B0F-FC50A85A23D1

Fig. 2b

Oestrus nasalis Linnaeus, 1758: 584. Type locality: Sweden.
Oestrus equi Fabricius, 1787: 321. Type locality: Probably Europe.
Oestrus veterinus Clark, 1797: 312. New replacement name for Oestrus nasalis Linnaeus, 1758.
Oestrus salutaris Clark, 1815: pl. 1. *Nomen nudum*.
Gasterophilus clarkii Leach, 1817: 2. Type locality: England (Bantham).
Gastrus jumentarum Meigen, 1824: 179. Type locality: Probably Denmark.
Oestrus gastricus minor Schwab, 1840: 40. Unavailable name.
Gastrus subjacens Walker, 1849: 687. Type locality: Canada (Nova Scotia).
Oestrus stomachinus Gistel, 1848: 153. Type locality: Probably Germany (Bavaria).
Gasterophilus crossi Patton, 1924: 963. Type locality: India (Punjab).
Gastrophilus albescens Pleske, 1926: 228. Type locality: Egypt (Cairo).
Gastrophilus nasalis var. nudicollis Dinulescu, 1932: 28, 32. Type locality: Unknown.
Gastrophilus veterinus var. aureus Dinulescu, 1938: 315. Type locality: Unknown.
Gastrus jumentorum : Brauer, 1863: 87, 280. Incorrect subsequent spelling of jumentarum Meigen, 1824.
Oestrus nasulis : Fabricius, 1787: 321. Incorrect subsequent spelling of nasalis Linnaeus, 1758.

#### Common name.

Throat bot fly or Horse nasal bot fly.

#### Distribution.

Cosmopolitan.

#### Localities, hosts, and dates of collection.

**Figure 4.**
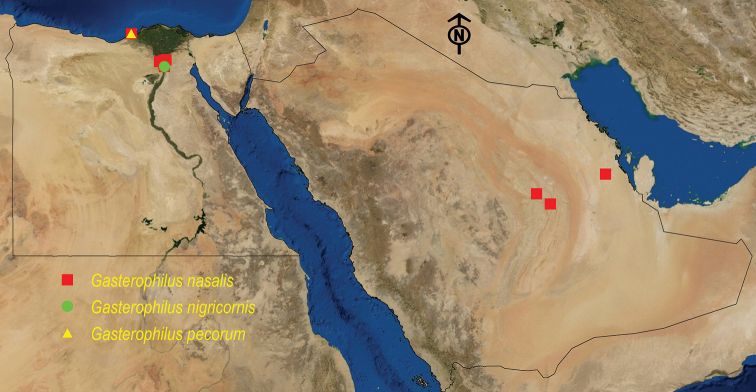
Distribution map of *G. nasalis*, *G. nigricornis*, and *G. pecorum*.

see Table 4 and Figure 4.

**Table 4.** Localities, hosts, and dates of collection of *G. nasalis*.

Country	Zone or Region	Locality	Coordinates	Host/s	Months of collection	Reference
Egypt	Coastal Strip	Alexandria	31.203358N, 29.917285E	mules and donkeys (from stomachs)	from October to April	El-Bakry and Fadly (2014)
	Lower Nile Valley & Delta	Abu-Rawash	30.045837N, 31.091406E	not given	May	museum material (see material examined)
		Cairo (at slaughter house of the National Cairo Circus)	30.122446N, 31.360598E	donkeys	throughout the year	Hilali et al. (1987)
		Cairo (no further data)	–	–	–	Li et al. (2019b)
		Cairo (at Cairo Manure Co.)	30.102160N, 31.253994E	mules (from stomachs)	June	museum material (see material examined)
		Helwan	29.839022N, 31.300160E	not given	April and December	museum material (see material examined)
		Maadi	29.961203N, 31.266910E	not given	April	museum material (see material examined)
KSA	Widespread in all regions, especially abundant in Al-Ehsaa, El-Kharj and Riyadh	Al-Ehsaa	25.388528N, 49.596223E	donkeys and horses (from stomachs)	March to September	Abu-Thuraya (1982)
		El-Kharj	24.148402N, 47.305011E	donkeys and horses (from stomachs)	March to September	
		Riyadh (near slaughterhouse)	24.578977N, 46.736175E	from dead domestic horse	March	Büttiker and Zumpt (1982)

#### Material examined.

Egypt • 1 male; Abu-Rawash; 30.045837N, 31.091406E; 18.May.1935; EFC • 1 female; Cairo Manure Co.; 30.102160N, 31.253994E; 11.Jun.1924; from the stomach of a mule; EFC • 1 male; Helwan; 29.839022N, 31.300160E; 18.May.1934 • 1 female; Maadi; 29.961203N, 31.266910E; 9.Apr.1916; EFC.

### Gasterophilus nigricornis

Taxon classificationAnimaliaDipteraOestridae

(Loew, 1863)

1C889187-11FA-5D16-887E-7F4ABF1C61F1

Fig. 2c

Gastrus nigricornis Loew, 1863: 38. Type locality: Moldova (Bessarabia).
Gastrophilus viridis Sultanov, 1951: 41. Type locality: Kazakhstan.
Gasterophilus migricornis : Colwell, 2006: 291. Incorrect subsequent spelling of nigricornis Loew, 1863.

#### Common name.

Horse stomach bot fly.

#### Distribution.

PA: China, Egypt, Kazakhstan, Kyrgyzstan, Moldova, Mongolia, Russia, Tajikistan, Turkmenistan, Ukraine, Uzbekistan (see Soós and Minar 1986a; Kettle 1995; Li et al. 2019b).

#### Localities, hosts, and dates of collection.

See Table 5 and Figure 4.

**Table 5.** Localities, hosts, and dates of collection of *G. nigricornis*.

Country	Zone or Region	Locality	Coordinates	Host/s	Months of collection	Reference
Egypt	Lower Nile Valley & Delta	Helwan	29.839022N, 31.300160E	not given	April	museum material (see material examined)

#### Material examined.

Egypt • 1 female; Helwan; 29.839022N, 31.300160E; 13.Apr.1935; EFC.

### Gasterophilus pecorum

Taxon classificationAnimaliaDipteraOestridae

(Fabricius, 1794)

9D609B46-4CB2-594D-BCE2-8571B3B7B255

Oestrus pecorum Fabricius, 1794: 230. Type locality: Probably Europe.
Oestrus vituli Fabricius, 1794: 231. Type locality: Not given, probably Sweden and France.
Gastrus jubarum Meigen, 1824: 179, 180. Type locality: Austria.
Gastrus lativentris Brauer, 1858b: 465. Type locality: Latvia (Curland).
Gastrus ferruginatus Zetterstedt, 1844: 978. Type locality: Sweden (Skåne, Tranås socken, Esperöd).
Gasterophilus pecorum var. zebrae Rodhain & Bequaert, 1920: 181. Type localities: Kenya and Tanzania.
Gastrophilus vulpecula Pleske, 1926: 227. Type locality: China (Inner Mongolia, Alxa League).
Gastrophilus gammeli Szilády, 1935: 140. Type locality: Hungary.
Gastrophilus hammeli : Paramonov, 1940: 34, 46. Incorrect subsequent spelling of gammeli Szilády, 1935.
Gastrus selysi Walker, 1849: 687. *Nomen nudum*.

#### Common name.

Dark-winged horse bot fly.

#### Distribution.

AF: Burkina Faso, Kenya, Namibia, Senegal, South Africa, Tanzania, Uganda, Zambia. OR: India. PA: Belgium, China (Heilongjiang, Inner Mongolia, Xinjiang), Czech Republic, Denmark, Egypt, France, Germany, Hungary, Iran, Italy, Latvia, Lithuania, Mongolia, Poland, Romania, Sweden, Switzerland, The Netherlands, Turkey, Ukraine, United Kingdom (see Soós and Minar 1986a; Kettle 1995; Li et al. 2019b).

#### Localities, hosts, and dates of collection.

See Table 6 and Figure 4.

**Table 6.** Localities, hosts, and dates of collection of *G. pecorum*.

Country	Zone or Region	Locality	Coordinates	Host/s	Months of collection	Reference
Egypt	Coastal Strip	Alexandria	31.203358N, 29.917285E	mules and donkeys (from stomachs)	from October to April	El-Bakry and Fadly (2014)

## Subfamily Hypodermatinae

### Genus Hypoderma

Taxon classificationAnimaliaDipteraOestridae

Latreille, 1818

E164938E-E52D-5E82-81CB-E25BAB11269C

Hypoderma Latreille, 1818: 272. Type species: Oestrus bovis Linnaeus, 1758, by monotypy.
Marmaryga Gistl, 1848: 9. Unjustified name for Hypoderma.
Atelecephala Townsend, 1916: 617. Type species: Hypoderma diana Brauer, 1858a, by monotypy.

### Hypoderma bovis

Taxon classificationAnimaliaDipteraOestridae

(Linnaeus, 1758)

47F461ED-719F-5920-8464-8FC0CBE72854

Oestrus bovis Linnaeus, 1758: 584. Type locality: Not given (? Sweden).
Oestrus ericetorum Clark, 1815. *Nomen dubium*.
Oestrus subcutaneus Greve, 1818: 2. Type locality: Not given.
Oestrus bovinus Schwab, 1840: 43. Type locality: Not given.
Hypoderma heteroptera Macquart, 1843: 181. Type locality: Algeria (Oran).
Hypoderma bellieri Bigot, 1862: 113. Type locality: France (Corsica).

#### Common name.

Ox warble fly.

#### Distribution.

AU: Hawaii, New Zealand. NE: Widespread. PA: Widespread.

#### Localities, hosts, and dates of collection.

Unknown.

#### Notes.

This species is known to be recorded in Egypt only from the list of Steyskal and El-Bialy (1967), but no specimens of this species were collected or found in the Egyptian museums.

### Hypoderma desertorum

Taxon classificationAnimaliaDipteraOestridae

Brauer, 1897

BB1C3D54-8A25-549C-AD71-F903349E180D

Hypoderma desertorum Brauer, 1897: 377. Type locality: Egypt (Helwan).

#### Common name.

No specific common name.

#### Distribution.

PA: Egypt.

#### Localities, hosts, and dates of collection.

**Figure 5.**
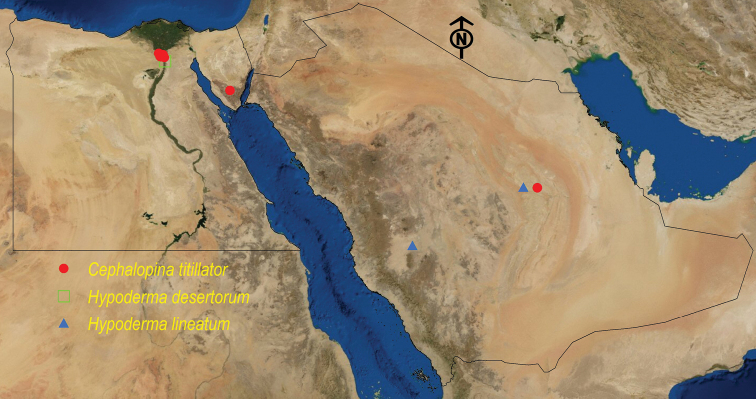
Distribution map of *C. titillator*, *H. desertorum*, and *H. lineatum*.

See Table 7 and Figure 5.

**Table 7.** Localities, hosts, and dates of collection of *H. desertorum*.

Country	Zone or Region	Locality	Coordinates	Host/s	Months of collection	Reference
Egypt	Lower Nile Valley & Delta	Helwan	29.839022N, 31.300160E	not given	April	Brauer (1897)

#### Notes.

Steyskal and El-Bialy (1967) listed this species as a junior synonym of *Hypoderma bovis* (Linnaeus, 1758); however, Soós and Minar (1986b) catalogued it as a valid species. No specimens are available to confirm its validity. Grunin (1965) keyed the *Hypoderma* spp. in the Palaearctic Region and used the colour of hairs on mesonotum, shape of antennal segments and body length to differentiated between *H. desertorum* and *H. bovis*. Holotype is deposited in Naturhistorisches Museum Wien, Wien, Austria (NMW).

### Hypoderma lineatum

Taxon classificationAnimaliaDipteraOestridae

(Villers, 1789)

199CCD0B-0C1C-5A9C-8FF2-DEA1B88BE394

Oestrus lineatum Villers, 1789: 349. Type locality: Not given (Europe).
Hypoderma bonassi Brauer, 1875: 75. Type locality: USA (Colorado).
Oestrus supplens Walker, 1849: 685. Type locality: Canada (Nova Scotia).

#### Common name.

Lesser cattle warble fly.

#### Distribution.

Cosmopolitan.

#### Localities, hosts, and dates of collection.

See Table 8 and Figure 5.

**Table 8.** Localities, hosts, and dates of collection of *H. lineatum*.

Country	Zone or Region	Locality	Coordinates	Host/s	Months of collection	Reference
KSA	Riyadh	Dhurma	24.613516N, 46.151759E	a dairy cow air-shipped from Canada	unknown	Büttiker and Zumpt (1982)
	Makkah	Wadi Qatan	22.200883N, 41.556635E	domestic goat	November	Büttiker and Zumpt (1982)

### Genus Przhevalskiana

Taxon classificationAnimaliaDipteraOestridae

Grunin, 1948

D3BA0FA4-E946-5634-BDFE-B01BC35DBEA5

Przhevalskiana Grunin, 1948: 469 (as subgenus of Hypoderma Latreille, 1818). Type species: Hypoderma orongonis Grunin, 1948, by monotypy.
Crivellia Grunin, 1956: 716. Type species: Hypoderma corinnae Crivelli, 1862, by original designation.

### Przhevalskiana silenus

Taxon classificationAnimaliaDipteraOestridae

(Brauer, 1858)

C7247CF7-A018-527A-9F0A-5302919A3DB8

Hypoderma silenus Brauer, 1858b: 460. Type localities: Italy (Sicily, Palermo); Egypt (Sinai).
Hypoderma aegagri Brauer, 1863: 134, 281. Type locality: Greece (Crete).
Hypoderma gazellae Gedoelst, 1916: 263. Type locality: Tanzania (Massai).
Hypoderma crossi Patton, 1922: 573. Type locality: India (Punjab).
Hypoderma aeratum Austen, 1931: 423. Type locality: Cyprus (Tillyria, Kyrenia).
Hypoderma capreum Gauser, 1940: 38. Type locality: Azerbaijan.

#### Common name.

Goat warble fly.

#### Distribution.

AF: East Africa, Saudi Arabia [as “South western part”]. OR: India. PA: Central Asia, Middle East, North Africa, southern Europe.

#### Localities, hosts, and dates of collection.

**Figure 6.**
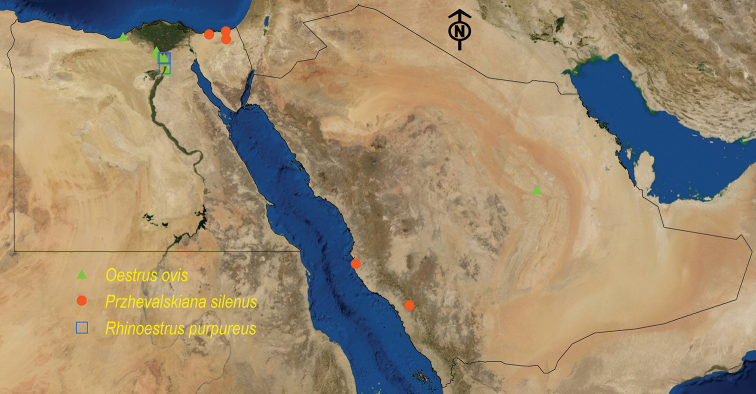
Distribution map of *O. ovis*, *P. silenus*, and *R. purpureus*.

See Table 9 and Figure 6.

**Table 9.** Localities, hosts, and dates of collection of *P. silenus*.

Country	Zone or Region	Locality	Coordinates	Hosts and/or methods of collection	Months of collection	Reference
Egypt	Sinai	Al Arish (abattoir)	31.131795N, 33.795749E	goats (larvae from slaughtered goats, and adults by baited traps)	throughout the year	Morsy et al. (1998)
		Bir Al Abd	31.005486N, 33.111721E	goats (larvae from slaughtered goats, and adults by baited traps)	throughout the year	Morsy et al. (1998)
		Hasanah	30.800220N, 33.815971E	goats (larvae from slaughtered goats, and adults by baited traps)	throughout the year	Morsy et al. (1998)
KSA	Al-Baha	Al-Mekhwa	19.759526N, 41.428219E	sweeping net by El-Hawagry	February	collected specimen (see material examined)
	Makkah	Jeddah (Jeddah Abattoir)	21.483464N, 39.201734E	goats (nodules caused by larvae are noticed on the backs of goat carcasses)	December to April	El-Azzazy (1997)

#### Material examined.

Saudi Arabia • 1 female; Al-Mekhwa; 19.759526N, 41.428219E; 3.Feb.2009; El-Hawagry leg.; sweeping net; MCCB.

## Subfamily Oestrinae

### Genus Cephalopina

Taxon classificationAnimaliaDipteraOestridae

Strand, 1928

42D54867-44FC-5D10-97C6-2B0FF01386A4

Cephalopina Strand, 1928: 48 (replacement name for Cephalopsis).
Cephalopsis Townsend, 1912: 53. Type species: Oestrus maculatus Wiedemann, 1830 (= Oestrus titillator Clark, 1816), by original designation. Preoccupied by Fitzinger, 1873 in Pisces.

### Cephalopina titillator

Taxon classificationAnimaliaDipteraOestridae

(Clark, 1816)

F4816E7E-68E9-5C96-9AEE-446AE5A8202D

Fig. 2d

Oestrus titillator Clark, 1816: 4. Type locality: Syria.
Oestrus maculatus Wiedemann, 1830: 256. Type locality: Egypt.
Oestrus libycus Clark, 1841: 100. *Nomen nudum*.
Oestrus libycus Clark, 1843: 93. Type locality: Egypt.
Pharyngobalus cameli Steel, 1887: 27. Type localities: Sudan, ?Afghanistan.

#### Common name.

Camel nasal bot fly.

#### Distribution.

AF: East Africa, Saudi Arabia [as “South western part”]. AU: Australia. OR: India. PA: Widespread in association with camels, particularly, Afghanistan, Middle East, Mongolia, North Africa, South Europe.

#### Localities, hosts, and dates of collection.

See Table 10 and Figure 5.

**Table 10.** Localities, hosts, and dates of collection of *C. titillator*.

Country	Zone or Region	Locality	Coordinates	Hosts and/or methods of collection	Months of collection	Reference
Egypt	Lower Nile Valley & Delta	Abu-Rawash	30.045837N, 31.091406E	dromedary camels (from the nasal cavities)	May	museum material (see material examined)
		Birqash	30.162842N, 31.039242E	sweeping, by El-Hawagry	June	collected specimens (see material examined)
		Cairo (Cairo abattoir)	30.040022N, 31.244248E	dromedary camels (from the nasal cavities)	throughout the year	museum material (see material examined)
		El-Bassatin (abattoir)	29.995917N, 31.276171E	camels	not given	Hendawy et al. (2012)
		El-Warrak (abattoir)	30.110544N, 31.210915E	camels	not given	Hendawy et al. (2012)
		Kerdassa	30.025663N, 31.113349E	dromedary camels (from the nasal cavities)	May	museum material (see material examined)
	Sinai	W. El-Sheikh	28.56568N, 33.96525E	not given	April	museum material (see material examined)
KSA	all regions	widespread	–	dromedary camels (nasal cavities)	throughout the year	Abu-Thuraya (1982); Alahmed (2002)
	Riyadh	Riyadh (slaughterhouse)	24.578977N, 46.736175E	dromedary camels	March to May	Büttiker and Zumpt (1982)
	Makkah	Jeddah (Jeddah abattoir)	21.483464N, 39.201734E	dromedary camels	throughout the year	Gadallah and Bosly (2006)

#### Material examined.

Egypt • 1 male; Cairo abattoir; 30.040022N, 31.244248E; 6.Jun.1924; Efflatoun leg.; from nose of camel; EFC • 1 male; same data as for preceding; 2.Jul.1924 • 1 female; same data as for preceding; 19.Nov.1929 • 1 male; Kerdassa; 30.02566N, 31.11335E; 19.May.1924; R.M. leg.; from nose of camel; EFC • 1 male, 1 female; Sinai, W. El-Sheikh; 28.56568N, 33.96525E; 21–27.Apr.1939; B.C.E. leg.; EFC • 1 female; Cairo abattoir; 30.040022N, 31.244248E; 20.Jan.1924; H.C.E. leg.; from the nose of a camel; PPDD • 1 female, 1 male; Birqash; 30.162842N, 31.039242E; 21.Jun.1999; El-Hawagry leg.; sweeping net; MSHC.

Saudi Arabia • 2 females; Riyadh, slaughterhouse; 24.578977N, 46.736175E; 30.Oct.1999; Azzam Alahmed leg.; from dromedary camels; KSMA.

### Genus Oestrus

Taxon classificationAnimaliaDipteraOestridae

Linnaeus, 1758

49C3240B-945C-5E3D-B28E-B0039DBAFF01

Oestrus Linnaeus, 1758: 584. Type species: Oestrus ovis Linnaeus, 1758, by original designation of Curtis, 1826: 106.
Cephalemyia Latreille, 1818: 273. Type species: Oestrus ovis Linnaeus, 1758, by monotypy.
Cephalomyia Agassiz, 1846: 71. Unjustified emendation of Cephalemyia.

### Oestrus ovis

Taxon classificationAnimaliaDipteraOestridae

(Linnaeus, 1758)

1E9C5CEC-FA50-5327-85FA-A90A423D3972

Oestrus ovis Linnaeus, 1758: 585. Type locality: Not given (? Sweden).
Oestrus argalis Pallas, 1776: 29. Type locality: Not given (? Middle Asia).
Oestrus perplexus Hudson, 1892: 63. Type locality: New Zealand. *Nomen nudum*.

#### Common name.

Sheep nasal bot fly.

#### Distribution.

Cosmopolitan (introduced with sheep in most parts of the world, see Papavero (1977)).

#### Localities, hosts, and dates of collection.

See Table 11 and Figure 6.

**Table 11.** Localities, hosts, and dates of collection of *O. ovis*.

Country	Zone or Region	Locality	Coordinates	Hosts and/or methods of collection	Months of collection	Reference
Egypt	Coastal Strip	Burg	30.916760N, 29.533268E	not given	March	material (see material examined)
	Eastern Desert	Wadi El-Mallah	–	not given	May	material (see material examined)
		Wadi Hoff	29.880357N, 31.312991E	not given	April	material (see material examined)
		Wadi Rishrash	29.41666N, 31.51666E	not given	November to April	material (see material examined)
	Lower Nile Valley & Delta	Ashmoun Gereiss	30.325046N, 30.925513E	sheep (reared from larvae from nose)	March	material (see material examined)
		Cairo, Cairo (abattoir)	30.040022N, 31.244248E	sheep (from nose)	April to December	museum material (see material examined) and Amin et al. (1997)
		El-Hager	30.282066N, 30.913711E	sweeping net by El-Hawagry	April	collected specimens (see material examined)
		El-Katta	30.225859N, 30.970563E	not given	September	museum material (see material examined)
		Kerdassa	30.025663N, 31.113349E	sheep (from nose)	March and April	museum material (see material examined)
		Wardan	30.321045N, 30.905128E	sheep (reared from larvae from nose)	March	material (see material examined)
KSA	all regions	widespread	–	sheep and goats (from the nasal cavities and head sinuses)	March to June	Abu-Thuraya (1982)
	Asir	widespread (slaughterhouses)	–	not given	throughout the year	Kenawy et al. (2014)
	Jazan	Abu Arish	16.9595N, 42.8348E	Sheep (heads)	throughout the year	Bosly (2013)
	Riyadh	Riyadh (slaughterhouse)	24.578977N, 46.736175E	sheep and goats	May	Büttiker and Zumpt (1982)

#### Material examined.

Egypt • 1 male; Burg; 30.916760N, 29.533268E; 16.Mar.1935; H.C.E & M.T leg.; EFC • 3 males, 3 females; Cairo, Cairo abattoir; 30.040022N, 31.244248E; 5.Jun.1929; Efflatoun leg.; from sheep’s nose; EFC • 1 male, 1 female; same data as for preceding; 23.Dec.1929 • 2 males; same data as for preceding; 26.Nov.1929 • 1 male, same data as for preceding; 2.Jul.1924 • 1 male, same data as for preceding; 2. Apr.1924 • 1 female, same data as for preceding; 5. Apr.1924 • 1 female; Kerdassa; 30.025663N, 31.113349E; 18.Mar.1924; from the nose of sheep; EFC • 1 female; same data as for preceding; 22.May.1924; R. M. leg. • 1 female; Wadi Hoff; 29.880357N, 31.312991E; 14.Apr.1921; Efflatoun leg.; EFC • 1 female; Wadi Rishrash; 29.41666N, 31.51666E; 16.Apr.1932; ET & R leg.; EFC • 1 female; Wadi Rishrash; 29.41666N, 31.51666E; 29.Mar.1935; H.C.E. & M.T. leg.; EFC • 1 male; Ashmoun Gereiss; 30.325046N, 30.925513E; Wardan; 30.321045N, 30.905128E; 23.Mar.1924; H.C.E. leg.; reared from larvae from the nose of sheep; PPDD • 1 female; El-Mallah, East of Helwan; 3.May.1926; Farag leg.; PPDD • 1 female; El-Katta; 30.225859N, 30.970563E; 20.Sep.1924; PPDD • 1 male; Kerdassa; 30.025663N, 31.113349E; 15.May.1938; Mabrouk leg.; PPDD.

### Genus Rhinoestrus

Taxon classificationAnimaliaDipteraOestridae

Brauer, 1886

39980F9B-D17E-5D37-A50A-1507F9EB930C

Rhinoestrus Brauer, 1886: 300. Type species: Cephalomyia purpurea Brauer, 1858, by monotypy.
Hippoestrus Townsend, 1933: 447. Type species: Rhinoestrus hippopotami Grünberg, 1904, by original designation.

### Rhinoestrus purpureus

Taxon classificationAnimaliaDipteraOestridae

(Brauer, 1858)

5B13BD52-F039-545A-AC84-3FFA45EE9758

Cephalomyia purpurea Brauer, 1858b: 457. Type locality: Austria (Bisamberg).
Rhinoestrus nasalis : Brumpt, 1913: 700. Misidentification.

#### Common name.

Equine nasal bot fly.

#### Distribution.

AF, OR: Widespread (introduced with horses, see Papavero (1977)). PA: Widespread.

#### Localities, hosts, and dates of collection.

See Table 12 and Figure 6.

**Table 12.** Localities, hosts, and dates of collection of *R. purpureus*.

Country	Zone or Region	Locality	Coordinates	Hosts and/or methods of collection	Months of collection	Reference
Egypt	Lower Nile Valley & Delta	Cairo	29.999896N, 31.270483E	Donkey (from head)	May	museum material (see material examined)
		El-Magadlah	–	not given	April	museum material (see material examined)
		Giza	30.015432N, 31.207837E	not given	May	museum material (see material examined)
		Giza, Giza zoo abattoir (donkeys originally obtained from four governorates: Giza, Monofia, Fayoum, and Bani Sweif)	30.027973N, 31.215963E	donkeys	throughout the year	Hilali et al. (2015)

**Material examined.** Egypt • 1 male; Cairo; 29.999896N, 31.270483E; 10.May.1922; Efflatoun leg.; from donkey’s head; EFC • 1 male; El-Magadlah; 27.Apr.1924; R. Mabrouk leg.; EFC • 1 female; Giza; 30.015432N, 31.207837E; 2.May.1907; EFC.

## Discussion

Egypt and Saudi Arabia are biogeographically comparable being located at the junction of the Palearctic and the Afrotropical Realms. In Egypt, the Afrotropical Realm is thought to involve the southeastern triangle of the country, which known as the Gebel Elba ecological zone. This is the only ecological zone in Egypt, which has an Afrotropical faunal affiliation. However, the faunal affiliation of the other seven ecological zones is mostly Palearctic, namely, the Coastal Strip, Eastern Desert, Western Desert, Fayoum, Lower Nile Valley, and Delta, Sinai, and Upper Nile Valley (Fig. 1) (El-Hawagry and Gilbert 2014; El-Hawagry 2017; El-Hawagry et al. 2018; El-Hawagry et al. 2020). In Saudi Arabia, many biogeographers agree that the border of the Afrotropical Realm should be extended up to Taif City, i.e., up to the Tropic of Cancer, covering the southwestern part of the country (Wallace 1876; Hölzel 1998; El-Hawagry et al. 2017; El-Hawagry and Al Dhafer 2019; El-Hawagry et al. 2019). All these biogeographic facts undoubtedly reflects on the distribution of oestrid species treated in the present study as all reported species, except three, are of both Palaearctic and Afrotropical affinities. Only *Gasterophilus nigricornis* and *Hypoderma bovis* are Palaearctic, and *Hypoderma desertorum* is endemic to Egypt. Some of the reported species are also known as cosmopolitan and should be widespread in both Egypt and Saudi Arabia; however, the majority of species were reported only from some restricted regions. Surprisingly, no records of oestrid flies were reported from Upper Nile Valley, Western Desert and Gebel Elba in Egypt. This is most likely due to the fact that most collections were focused predominantly in Alexandria, Greater Cairo (slaughterhouses, circus, Giza Zoo, Manure Co., near pyramids and wadies southwestern to Cairo) and Sinai Peninsula. The same situation is in Saudi Arabia as few records were reported especially from Al-Baha, Eastern Province, Makkah, and Riyadh regions (Abu-Thuraya 1982).

Oestrid flies in Egypt and Saudi Arabia, as far as is known, infest domesticated animals and in some cases humans. Infections with *Cephalopina titillator* larvae have been reported in the dromedary camel (Family Camelidae) (Abu-Thuraya 1982, Büttiker and Zumpt 1982, Hussein et al. 1982, Fatani and Hilali 1994, Alahmed 2002, Hendawy et al. 2012). Attacks by larvae of different *Gasterophilus* species have been reported in donkeys and horses (family Equidae) (Abu-Thuraya 1982, Büttiker and Zumpt 1982, Hilali et al. 1987, El-Bakry and Fadly 2014, Abdel Rahman et al. 2018, Attia et al. 2018) and *Rhinoestrus purpureus* (Hilali et al. 2015). The goats and sheep (Family Bovidae) have been reported as hosts for the larvae of *Hypoderma lineatum* (Büttiker and Zumpt 1982), *Oestrus ovis* (Abu-Thuraya 1982, Büttiker and Zumpt 1982, Amin et al. 1997, Bosly 2013), and *Przhevalskiana silenus* (El-Azzazy 1997, Morsy et al. 1998). Ophthalmomyiasis infestation of human eye with larvae of *O. ovis* was documented from Saudi Arabia (Omer et al 1988). Two cases of gastric myiasis with larvae of unidentified *Oestrus* sp. were reported from Egypt, Minia Governorate (Ahmad et al. 2011).

The low abundance and diversity of species in both Egypt and Saudi Arabia should be taken with caution, since the family seems to lack sampling efforts in both countries. We think that the distributional data of these economically important flies within Egypt and Saudi Arabia is still scanty, and more efforts would be highly desirable in the future. Nevertheless, the present catalogue presented some new locality records especially for *Gasterophilus intestinalis*, *Gasterophilus nasalis*, *Gasterophilus nigricornis*, *Przhevalskiana silenus*, *Cephalopina titillator*, *Oestrus ovis* and *Rhinoestrus purpureus*. This catalogue undoubtedly will act as a baseline for further study in both countries.

## Supplementary Material

XML Treatment for Gasterophilus

XML Treatment for Gasterophilus haemorrhoidalis

XML Treatment for Gasterophilus intestinalis

XML Treatment for Gasterophilus nasalis

XML Treatment for Gasterophilus nigricornis

XML Treatment for Gasterophilus pecorum

XML Treatment for Hypoderma

XML Treatment for Hypoderma bovis

XML Treatment for Hypoderma desertorum

XML Treatment for Hypoderma lineatum

XML Treatment for Przhevalskiana

XML Treatment for Przhevalskiana silenus

XML Treatment for Cephalopina

XML Treatment for Cephalopina titillator

XML Treatment for Oestrus

XML Treatment for Oestrus ovis

XML Treatment for Rhinoestrus

XML Treatment for Rhinoestrus purpureus
